# Evaluation of Safety, Immunogenicity, and Protective Efficacy of an Orally Administered African Swine Fever Vaccine Candidate ASFV-G-∆I177L/∆LVR

**DOI:** 10.3390/vaccines14070609

**Published:** 2026-07-10

**Authors:** Yeonji Kim, Sun A. Choi, Wonjun Kim, Yongwoo Shin, Sua Choi, Ji-yun Sung, So-Jeong Kim, Seong Cheol Moon, Su Jin Lee, Xinghua Zheng, Se Young Lee, Keun Seung Ahn, Dongseob Tark, Jung Hyang Sur, Weonhwa Jheong

**Affiliations:** 1 Wildlife Disease Response Team, National Institute of Wildlife Disease Control and Prevention (NIWDC), Ministry of Climate, Energy and Environment, Gwangju 62407, Republic of Korea; yeonchi16@korea.kr (Y.K.); kwj22081@korea.kr (W.K.); jams80@korea.kr (Y.S.); choisua@korea.kr (S.C.); jo8261@korea.kr (J.-y.S.); th3555@korea.kr (S.-J.K.); 2 College of Veterinary Medicine, Chungnam National University, Daejeon 34134, Republic of Korea; 3 Central Research and Development Institute, Komipharm International Co., Ltd., Siheung-si 15094, Republic of Korea; dktjschl@komipharm.com (S.A.C.); moonvet@komipharm.com (S.C.M.); leesoot@komipharm.com (S.J.L.); xinghua@komipharm.com (X.Z.); man32@komipharm.com (S.Y.L.); pig182@komipharm.com (K.S.A.); 4 Department of Veterinary Pathology, College of Veterinary Medicine, Chonnam National University, Gwangju 61186, Republic of Korea; 5 Laboratory for Infectious Disease Prevention, College of Veterinary Medicine, Korea Zoonosis Research Institute, Jeonbuk National University, Iksan 54531, Republic of Korea; tarkds@jbnu.ac.kr

**Keywords:** African swine fever virus, live-attenuated vaccine, oral administration, dose evaluation, protective efficacy

## Abstract

**Background/Objective:** African swine fever (ASF), caused by African swine fever virus (ASFV), is a highly contagious viral disease affecting domestic pigs and wild boars, causing severe economic losses. Although commercial ASF vaccines have recently been approved in Vietnam, controlling ASF transmission remains challenging. Since injection-based vaccination is impractical for wild boars, oral vaccination is considered essential. This study aimed to evaluate the safety, immunogenicity, protective efficacy, and dose-related outcomes of ASFV-G-ΔI177L/ΔLVR, a live attenuated ASFV vaccine candidate with deletion in the *I177L* gene and left variable region (LVR), administered orally to convention pigs. **Methods:** The ASFV-G-ΔI177L/ΔLVR vaccine candidate was orally administered to conventional pigs at three dose levels (10^2.25^, 10^5.0^, and 10^6.0^ TCID_50_/dose). At 28 days post-vaccination, pigs were challenged intramuscularly with a virulent ASFV field strain at 10^2.0^ HAD_50_/mL and monitored clinically. Protection was assessed by ASFV-specific antibody responses (p32) and survival following challenge. **Results:** Oral immunization was well tolerated, with no vaccine-associated clinical signs observed before challenge. Following challenge, vaccinated pigs showed different protective outcomes among the tested dose groups, with survival rates of 1/4 (10^2.25^ TCID_50_/dose), 4/4 (10^5.0^ TCID_50_/dose), and 3/4 (10^6.0^ TCID_50_/dose), respectively. Pigs that succumbed to infection showed neither detectable viremia nor ASFV-specific antibodies before challenge, suggesting incomplete vaccine uptake may have resulted in insufficient immune induction rather than an adverse effect associated with vaccination. In contrast, pigs that seroconverted prior to challenge were fully protected and exhibited lower viral loads than the control animals. **Conclusions:** ASFV-G-ΔI177L/ΔLVR was well tolerated as an oral live attenuated vaccine candidate and induced protective immunity against virulent ASFV challenge under the present experimental conditions. Notably, complete protection was observed in the 10^5.0^ TCID_50_/dose group, supporting the potential of this vaccine candidate for oral immunization strategies against ASF. However, the present data do not allow a definitive conclusion regarding the dose–response relationship, and further studies with larger group sizes and field-relevant models are needed to refine dose selection and practical applicability, particularly for wild boar vaccination.

## 1. Introduction

African swine fever (ASF), classified as a notifiable disease by the World Organization for Animal Health (WOAH), caused by African swine fever virus (ASFV), is affecting wild boars and domestic pigs globally. It has affected countries across Eurasia and spreads geographically, causing significant damage to the pig production industry [1,2].

In Asia, no countries, except those in Russia, had experienced ASF outbreaks prior to 2018. Since the first outbreak, which was reported in China [3], ASF has spread rapidly in Asia including countries such as Mongolia, Vietnam, Cambodia, North Korea, Laos, Philippines, Myanmar, Russia, South Korea, and Timor-Leste in 2019 [4]. The first ASF case in Korea was reported at a pig farm in Paju [5]. The first wild boar ASF case in Korea was reported in Yeoncheon [6]. Following this, the outbreak was limited to Yeoncheon, Cheolwon, and Hwacheon, but since 2020, ASF has spread to the east and south.

Various efforts are being made to prevent the spread of the ASF in South Korea, including searching and removing carcasses, reducing the density of wild boars and blocking the route using fences. Nevertheless, an effective and safe vaccine is essential for preventing ASF [7].

Recent research has focused on the development of vaccines against ASFV, including inactivated vaccines, recombinant subunit vaccines, vector-based vaccines, and live attenuated vaccine (LAV) candidates. Among these, LAVs have shown the most consistent protective efficacy in clinical trials and are currently considered the most promising approach for preventing ASFV infection [7,8,9,10]. However, despite their efficacy, concerns regarding safety and limited data on long-term effects remain major obstacles to their widespread deployment [11].

Considering the ongoing spread of ASFV and limitations of current control strategies, there is an urgent need to develop practical vaccine platforms for wild boar populations, which act as reservoirs of the virus in endemic areas. Oral administration is considered a promising strategy for wild boar vaccination because it enables vaccine delivery without the need for direct capture or individual injection [12,13,14]. However, the efficacy of orally administered ASFV vaccine candidates can be affected by vaccine dose, formulation, delivery method, and individual variation in oral uptake. Therefore, controlled experimental studies in pigs are needed as an initial step to evaluate the safety, immunogenicity, and protective potential of oral vaccination before field-oriented applications are considered.

In this study, we evaluated the safety, immunogenicity, protective efficacy, and dose-related outcomes of orally administered ASFV-G-ΔI177L/ΔLVR, a live attenuated oral vaccine candidate, in domestic pigs. The vaccine was administered as a liquid virus suspension at three different doses, and vaccinated pigs were subsequently challenged with a virulent Korean ASFV isolate under controlled experimental conditions. This study was designed to assess whether oral administration of ASFV-G-ΔI177L/ΔLVR could induce protective immunity and to provide preliminary information for further development of oral ASF vaccination strategies.

## 2. Materials and Methods

### 2.1. Cell Culture and Viruses

To prepare a master seed virus suitable for subsequent experimental use, the vaccine virus was propagated in cell culture with consideration of viral growth efficiency, genetic stability during passage, and final virus yield. Virus preparation was therefore performed in accordance with WOAH-recommended quality considerations.

The vaccine candidate strain ASFV-G-ΔI177L/ΔLVR was originally obtained from the United States Department of Agriculture as a passage 8 (P8) live attenuated ASF vaccine virus grown in Plum Island porcine epithelial cells (PIPECs) [15]. PIPECs, derived from porcine fetal kidney cells, stably express bovine αVβ6 integrin. Cells were maintained in Dulbecco’s Modified Eagle Medium (Gibco, Grand Island, NY, USA) supplemented with 10% fetal bovine serum (HyClone Laboratories, Logan, UT, USA), 1% penicillin–streptomycin (Gibco, Grand Island, NY, USA), and 2 mM L-glutamine. Cell cultures were incubated at 37 °C in a humidified atmosphere containing 5% CO_2_ [15].

After receiving the P8 virus, the virus was passaged once to obtain P9 and subsequently subjected to additional serial passages in PIPECs. Cell culture passages were carried out at 7–10 day intervals and mCherry expression was monitored by fluorescence microscopy. Among the serially propagated virus stocks, the preparation showing the highest infectious titer was selected as the master seed virus and designated ASFV-G-ΔI177L/ΔLVR-P19. The selected virus stock was further confirmed by genetic characterization [7,16].

Virus infectivity titers were measured using endpoint dilution assays. The titer of the ASFV-G-ΔI177L/ΔLVR vaccine virus was determined in PIPECs and expressed as TCID50, with values calculated according to the Spearman–Kärber method. In contrast, the titer of the virulent challenge virus was determined in primary swine macrophage cultures and expressed as HAD_50_, with values calculated by the Reed–Muench method based on hemadsorption activity [17].

### 2.2. Next-Generation Sequencing of ASFV Genomes

#### 2.2.1. Viral DNA Extraction for Next-Generation Sequencing Analysis

Total DNA was extracted directly from 300 μL of ASFV-G-ΔI177L/ΔLVR using the Maxwell RSC Whole blood DNA Kit (Promega Corporation, Madison, WI, USA) according to the manufacturer’s instructions.

#### 2.2.2. Complete Genome Sequencing of ASFV

Genomic DNA was fragmented and used for library construction with the Enzymatic Preparation Kit (Celemics, Inc., Seoul, Republic of Korea) according to the manufacturer’s instruction. The prepared gDNA libraries were then hybridized with ASFV-specific capture probes using the Celemics target genomic regions. The capture probes were designed and chemically synthesized to specifically hybridize to the ASFV target regions. After target capture, the enriched libraries were amplified by post-capture PCR to obtain sufficient library yield for sequencing.

The target-enriched libraries were sequenced on an Illumina NextSeq550 instrument (Illumina, Inc., San Diego, CA, USA) using a paired-end 2 × 150 bp read configuration. The raw sequencing data were obtained as FASTQ files. The paired-end reads were interleaved and quality-trimmed using BBTools v39.75 to remove low-quality sequences. The quality of the trimmed reads was assessed using FastQC v0.12.1, and the quality control results were summarized using MultiQC v1.33. The processed reads were then mapped to the reference ASFV genome (accession number: MW701371.1) using BWA-MEM v0.7.17. The resulting alignment files were processed using SAMtools v1.23.

#### 2.2.3. Genetic Stability Analysis

To assess the genetic stability of the vaccine candidate during serial passages, whole-genome sequencing data from representative passage samples (P19, P24, and P29) were analyzed relative to the reference genome (MW701371.1). Single nucleotide polymorphisms and insertion–deletion mutations were detected from the mapped sequencing reads. Variant calling was performed using BCFtools v1.23, followed by variant filtering using BCFtools v1.23. The filtered variants were annotated using custom Python scripts implemented in Python 3.13.11 with Biopython 1.86. The presence, number, and genomic locations of detected variants were compared among passage samples to evaluate whether sequence variation progressively accumulated during serial passage.

### 2.3. Animal Experiments

Animal experiments were performed using commercial pigs. All the animal experiments were performed under Animal Biosafety Level 3 conditions at the Korea Zoonosis Research Institute, Jeonbuk National University in Iksan, Republic of Korea, following a protocol approved by the Institutional Animal Care and Use Committee (protocol JBNUNON 01-022-2023 and JBNUNON 2024-049) in compliance with the Animal Welfare Act.

The protective efficacy of ASFV-G-ΔI177L/ΔLVR was assessed in ASFV-free and antibody-seronegative pigs (7–8 weeks). Each group included four pigs. The pigs were acclimatized for 1 week before the start of the experiment and managed with appropriate feeding and water supply systems, cleaning, and general veterinary care. Each experimental group was located in a separate isolator.

The vaccine candidate was prepared and administered as a liquid virus suspension. No bait-based formulation was used in this study. For oral vaccination, each pig received 5 mL of the liquid vaccine suspension containing the assigned dose of ASFV-G-ΔI177L/ΔLVR (Passage 19). The vaccine suspension was administered directly into the oral cavity using a sterile syringe without a needle, and care was taken to ensure that the inoculum was swallowed.

**Figure 1:**
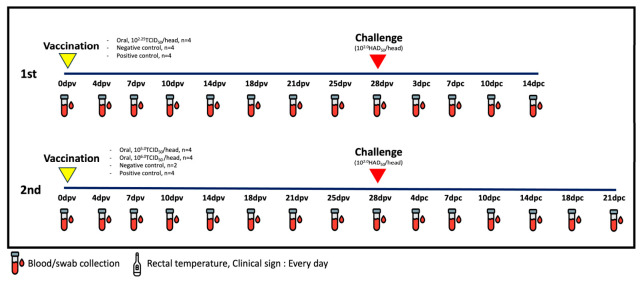
Experimental design for evaluating the efficacy of oral African swine fever virus (ASFV) vaccine in pigs. Yellow triangle: oral vaccination (ASFV-G-ΔI177L/ΔLVR); red triangle: challenge with virulent ASFV (Hwacheon/2020); dpv, day post vaccination; dpc, day post challenge.

The first experiment (1-A) was conducted to test the efficacy and safety of low dose ASFV-G-∆I177L/∆LVR, while the second experiment (2-A, 2-B) tested the efficacy and safety of medium and high doses of the same vaccine strain. Whole blood, serum, rectal swab, and oral swab samples were collected during the post-vaccination and post-challenge periods according to the sampling schedule. Twenty-eight days after vaccination, the pigs were challenged intramuscularly with a virulent Korean ASFV isolate (ASFV/Hwacheon/2020; GenBank accession no. OR159219.1). Whole-blood and swab samples were used for ASFV genomic DNA detection by qPCR, whereas serum samples were used for ASFV-specific antibody detection (Table 1, Figure 1).

Clinical signs were retrospectively scored using a semi-quantitative scoring system based on the recorded daily observations and previously described criteria [18]. The evaluated parameters included fever, liveliness, body shape, breathing, neurological signs, skin changes, digestive symptoms, and ocular/nasal discharge. Gross pathological changes observed at necropsy were also semi-quantitatively scored according to the severity of macroscopic lesions in major organs and based on previously described criteria [18]. The evaluated categories included body condition, integument, cardiovascular changes, liver, lung, spleen, lymph nodes/tonsil, and kidney. Each category was scored from 0 to 3 according to lesion severity, with 0 indicating no gross lesion and 3 indicating severe gross pathological changes.

### 2.4. Quantitative Real-Time PCR for the Detection of ASFV p72 and I177L Genes

Total DNA was extracted directly from 300 μL of EDTA-treated whole-blood, rectal swab, and oral swab samples using the Maxwell RSC Whole Blood DNA Kit (Promega, USA), following the manufacturer’s instructions.

Detection of the *p72 gene* was performed using the VetMAX ASFV Detection Kit (Thermo Fisher Scientific, Waltham, MA, USA), according to the manufacturer’s protocol. The reaction conditions were as follows: one cycle at 50 °C for 2 min, one cycle at 95 °C for 10 min, and 45 cycles at 95 °C for 15 s and 60 °C for 60 s. Real-time PCR was performed using a QuantStudio 5 Real-Time PCR Instrument (Applied Biosystems, Thermo Fisher Scientific, Waltham, MA, USA).

Detection of the *I177L* gene was performed according to a previously reported protocol [19] using primer and probe sets designed for the *I177L*-specific qPCR assay (Table S1). Because the ASFV-G-ΔI177L/ΔLVR vaccine strain lacks the *I177L* gene, *I177L*-specific qPCR was used to determine whether the detected ASFV DNA originated from the vaccine strain or the virulent challenge virus.

Although the amplification protocols differed between the *p72* and *I177L*-specific qPCR assays, the same cutoff value was applied for interpretation. For both assays, Ct values < 40 were interpreted as positive, whereas Ct values ≥ 40 or undetermined Ct values were considered negative or below the detection limit.

### 2.5. Enzyme-Linked Immunosorbent Assay

ASFV-specific antibody titers were assessed using the ID Screen^®^ African Swine Fever Competition ELISA Kit (Innovative Diagnostics SAS, Grabels, France), according to the manufacturer’s guidelines. Before use in the experiments, the serum was inactivated at 56 °C for 1 h. The optical density (OD) was measured at a wavelength of 450 nm using a Multiskan SkyHigh Microplate Spectrophotometer (Thermo Fisher Scientific Oy, Vantaa, Finland). The signal-to-noise ratio (S/N%) was calculated as the ratio of the sample signal to the negative control signal, expressed as a percentage. S/N% was calculated using the following formula:

S/N% = ODsample−ODpositivecontrol ODnegativecontrol −ODpositivecontrol ×100

Serum samples with S/N% ≤ 40% were considered positive. In contrast, serum samples with S/N% ≥ 50% were considered uncertain and their analysis was repeated.

### 2.6. Pathological Examination and Histopathology

Major organs including the spleen, liver, kidney, lung, heart, and lymph nodes were collected for pathological examination. Lymph node samples were fixed in 10% neutral- buffered formalin, processed routinely, embedded in paraffin, sectioned, and stained with hematoxylin and eosin for histopathological evaluation.

### 2.7. DNA Extraction and qPCR Detection of ASFV Genomic DNA in Tissue Homogenates

For organ viral DNA detection, tissue samples collected at necropsy were homogenized to prepare 10% (*w*/*v*) tissue homogenates, and total DNA was extracted from 300 μL of the homogenates using the Maxwell RSC Whole Blood DNA Kit (Promega, USA) according to the manufacturer’s instructions. ASFV genomic DNA in tissue homogenates was detected by *p72* qPCR as described above.

### 2.8. Statistical Analysis

Graphs were generated using GraphPad Prism version 8.4.2 (GraphPad Software, San Diego, CA, USA). Due to the limited sample size in each experimental group, the results were interpreted descriptively.

## 3. Results

### 3.1. Genetic Stability of ASFV-G-ΔI177L/ΔLVR During Serial Passage

Whole-genome sequencing of serially passaged vaccine virus samples revealed only limited sequence variation across the analyzed passage range. The number of SNPs remained low and did not progressively increase from P19 to P29. Coding-region variants were detected in a limited number of genes and consisted mainly of nonsynonymous substitutions, with a smaller number of synonymous substitutions. No INDELs were identified in any analyzed passaged sample. These findings suggest that ASFV-G-ΔI177L/ΔLVR maintained overall genetic stability during the analyzed serial passage range (Table 2 and Table S2).

### 3.2. Evaluation of Immunogenicity, Protective Efficacy, and Pathology Following Oral Vaccination

#### 3.2.1. Low-Dose Oral Vaccination Study (10^2.25^ TCID_50_)

**Figure 2:**
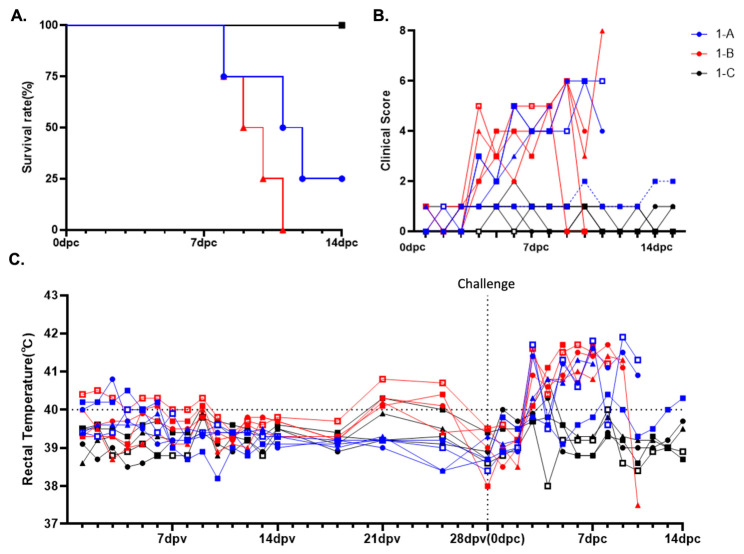
Survival, clinical scores, and rectal temperature in pigs orally vaccinated with ASFV-G-ΔI177L/ΔLVR (10^2.25^ TCID_50_/dose) and challenged with virulent ASFV-Hwacheon/2020 at 28 dpv. (**A**) Survival rate after challenge. (**B**) Daily clinical scores after challenge. (**C**) Rectal temperatures during vaccination and after challenge. The dashed line indicates the fever threshold (40 °C), and the dotted vertical line indicates the time of challenge. Blue, red, and black lines represent groups 1-A, 1-B, and 1-C, respectively, whereas different symbol shapes, fill patterns, and line styles distinguish individual animals within each group.

Group 1-A was orally immunized with a live attenuated ASFV-G-ΔI177L/ΔLVR vaccine at 10^2.25^ TCID_50_/dose to evaluate the safety and efficacy of the vaccine. At 28 dpv, pigs were intramuscularly challenged with 1 mL of a highly virulent homologous ASFV field strain (Hwacheon/2020) at a dose of 10^2.0^ HAD_50_/mL. Protective efficacy was defined as survival without severe clinical signs and maintenance of normal body temperature following challenge with a virulent ASFV strain. Unusual changes in body temperature were not observed in any animals prior to challenge. In contrast to a previous study involving intramuscular administration of the same strain [7], which conferred complete protection with only transient fever and no mortality, oral administration at the same dose (10^2.25^ TCID_50_) failed to provide full protection. Three out of four animals exhibited elevated body temperatures (40.3–41.9 °C) following the challenge and subsequently succumbed to infection, whereas only one animal maintained a normal temperature and survived (Figure 2). These findings suggest that the oral route at this dose may be insufficient to confer protective immunity.

**Figure 3:**
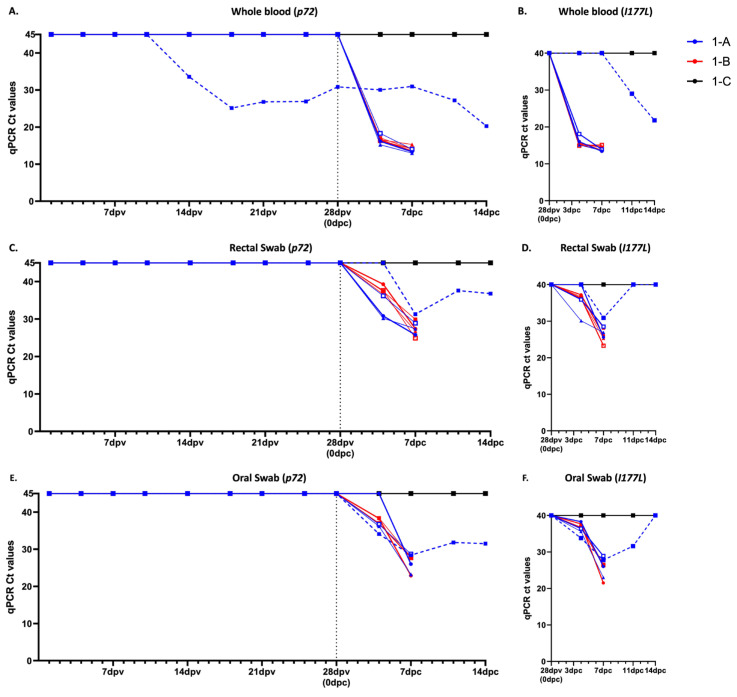
qPCR Ct values for ASFV genomic DNA detection and *I177L*-specific differential detection in whole blood and swab samples from pigs orally vaccinated with 10^2.25^ TCID_50_/dose ASFV-G-ΔI177L/ΔLVR in the first trial and challenged with ASFV-Hwacheon/2020. (**A**,**C**,**E**) *p72* qPCR and (**B**,**D**,**F**) *I177L*-specific qPCR results in whole blood, rectal swab, and oral swab samples, respectively. Blue, red, and black lines represent groups 1-A, 1-B, and 1-C, respectively. Different symbol shapes and fill patterns distinguish individual animals within each group. Each line represents an individual animal. Given the small number of animals per group, data are shown as individual longitudinal profiles rather than group-level statistical summaries. The vertical dotted line indicates the day of challenge, corresponding to 28 days post-vaccination (dpv) and 0 days post-challenge (dpc). The dashed animal line indicates the only surviving animal in the 10^2.25^ TCID_50_/dose group after challenge. For *p72* qPCR, Ct values < 40 were interpreted as ASFV DNA detected in this study, whereas Ct values ≥ 40 were considered negative or below the detection limit. A Ct value of 45 indicates no detectable amplification within 45 cycles. For *I177L* qPCR, Ct 40 was considered negative or below the detection limit. Lower Ct values indicate higher levels of viral DNA. *I177L*-specific qPCR was used to distinguish challenge virus-derived viral DNA from vaccine-derived viral DNA.

In whole-blood samples, *p72*-targeted ASFV genomic DNA was detected in only one animal during the vaccination period, with Ct values from 25.13 to 33.57. Notably, this animal survived the subsequent challenge (Figure 3A; Table S3). In rectal and oral swab samples, *p72*-targeted ASFV genomic DNA was not detected during the vaccination period in Group 1-A animals, whereas viral DNA was detected after challenge, particularly in animals that succumbed to infection (Figure 3C,E; Tables S4 and S5). *I177L*-specific qPCR was negative at 28 dpv, immediately before challenge, including in the animal with detectable *p72*-positive ASFV DNA in whole blood, supporting that pre-challenge *p72* detection was associated with the *I177L*-deleted vaccine strain. After challenge, *I177L*-positive signals were detected in whole-blood and/or swab samples. However, the surviving vaccinated animal showed delayed *I177L* detection and higher Ct values during the early post-challenge period compared with non-surviving vaccinated animals and positive-control animals, suggesting a lower early challenge virus-derived DNA burden (Figure 3B,D,F; Table S6).

**Figure 4:**
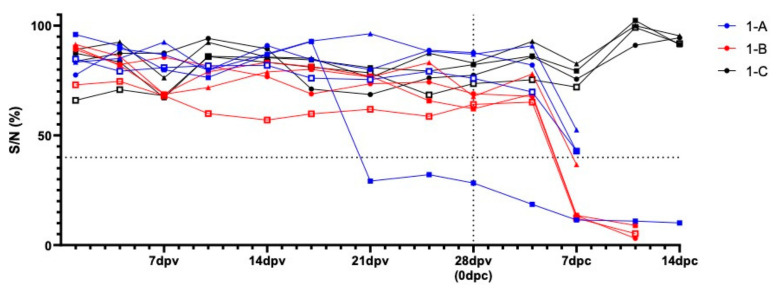
ASFV-specific antibody responses in serum samples from pigs in the first trial (10^2.25^ TCID_50_/dose). Pigs were orally vaccinated with 10^2.25^ TCID_50_/dose and subsequently challenged with ASFV-Hwacheon/2020 at 28 dpv. Blue, red, and black lines represent groups 1-A, 1-B, and 1-C, respectively. Different symbol shapes and fill patterns distinguish individual animals within each group. Each line represents an individual animal. The vertical dotted line indicates the day of challenge, corresponding to 28 dpv and 0 dpc. S/N values ≤ 40% were considered positive.

Seroconversion was also observed in this animal, as ASFV-specific p32 antibody levels exceeded the positive cutoff at 21 dpv (Figure 4; Table S7). In contrast, the remaining three animals showed neither detectable *p72*-targeted ASFV genomic DNA in whole-blood samples nor antibody responses during the vaccination period, suggesting a failure of vaccine uptake.

#### 3.2.2. Intermediate and High-Dose Oral Vaccination Study (10^5.0^ and 10^6.0^ TCID_50_)

To evaluate safety at higher oral doses, clinical monitoring was performed for 28 days after vaccination. In the 10^5.0^ TCID_50_/dose group (2-A), only one animal exhibited a mild, transient fever (40.3 °C) on 1 dpv, which resolved without further clinical signs. In contrast, all animals in the 10^6.0^ TCID_50_/dose inoculated group (2-B) showed elevated body temperatures (40.2–40.9 °C) on 1 dpv. One of them showed a further transient peak (40.5 °C) on 3 dpv, but all temperatures returned to normal thereafter. No sustained fever or vaccine-related clinical abnormalities were observed in either group during the vaccination period.

**Figure 5:**
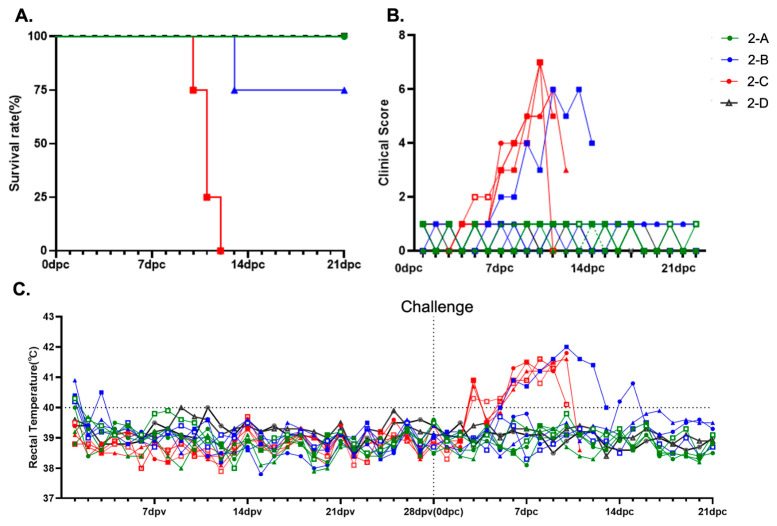
Survival, clinical scores, and rectal temperature in pigs orally vaccinated with ASFV-G-ΔI177L/ΔLVR at 10^5.0^ and 10^6.0^ TCID_50_/dose and challenged with virulent ASFV-Hwacheon/2020 at 28 dpv. (**A**) Survival rate after challenge. (**B**) Daily clinical scores after challenge. (**C**) Rectal temperature of pigs throughout ASF vaccination and challenge. During the vaccination phase, rectal temperatures were measured daily for the first 14 dpv and twice weekly thereafter until challenge. Following the challenge, rectal temperatures were recorded daily to monitor clinical progression. Green, blue, red, and gray lines represent groups 2-A, 2-B, 2-C, and 2-D, respectively. Different symbol shapes and fill patterns distinguish individual animals within each group, and each line represents an individual animal. The vertical dotted line indicates the day of challenge, corresponding to 28 dpv and 0 dpc. The horizontal dotted line indicates the fever threshold of 40 °C.

Following intramuscular challenge with a virulent ASFV strain at 28 dpv, all animals in both groups were closely monitored for clinical signs. All animals in Group 2-A maintained stable body temperatures throughout the post-challenge observation period, with no clinical abnormalities observed. In Group 2-B, three out of four animals also showed stable temperature profiles and survived without notable clinical signs. However, one animal in this group exhibited a sustained increase in body temperature, reaching 40.0 °C at 5 dpc and remaining elevated until euthanasia at 13 dpc (Figure 5).

**Figure 6:**
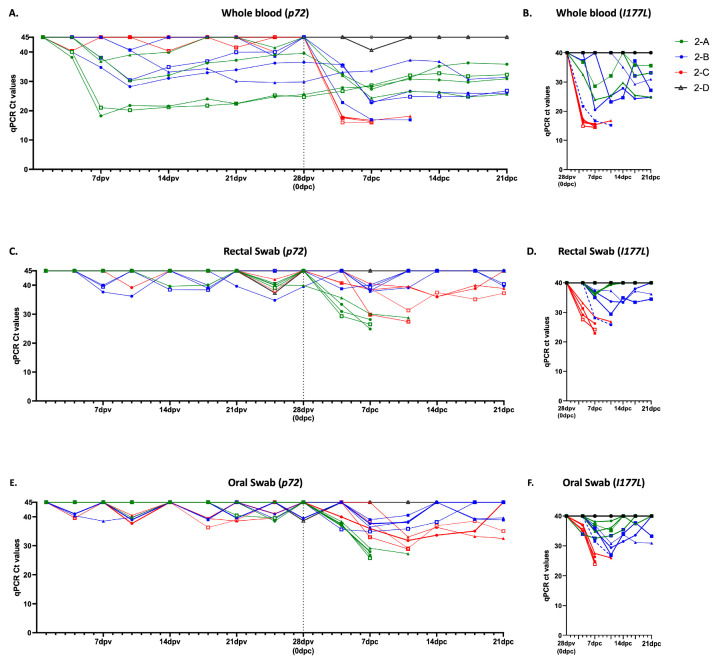
qPCR Ct values for ASFV genomic DNA detection and *I177L*-specific differential detection in whole blood and swab samples from pigs orally vaccinated with ASFV-G-ΔI177L/ΔLVR at 10^5.0^ or 10^6.0^ TCID_50_/dose in the second trial and subsequently challenged with ASFV-Hwacheon/2020. (**A**,**C**,**E**) *p72* qPCR and (**B**,**D**,**F**) *I177L*-specific qPCR results in whole blood, rectal swab, and oral swab samples, respectively. Green, blue, red, and black lines represent groups 2-A, 2-B, 2-C, and 2-D, respectively. Different symbol shapes and fill patterns distinguish individual animals within each group, and each line represents an individual animal. Given the small number of animals per group, data are shown as individual longitudinal profiles rather than group-level statistical summaries. The vertical dotted line indicates the day of challenge, corresponding to 28 days post-vaccination (dpv) and 0 days post-challenge (dpc). The dashed animal line indicates the single animal in the 10^6.0^ TCID_50_/dose group that succumbed after challenge. For *p72* qPCR, Ct values < 40 were interpreted as ASFV DNA detected in this study, whereas Ct values ≥ 40 were considered negative or below the detection limit. A Ct value of 45 indicates no detectable amplification within 45 cycles. For *I177L* qPCR, Ct 40 was considered negative or below the detection limit. Lower Ct values indicate higher levels of viral DNA. *I177L*-specific qPCR was used to distinguish challenge virus-derived viral DNA from vaccine-derived viral DNA.

In whole-blood samples, *p72*-targeted ASFV genomic DNA was detected in all Group 2-A animals during the vaccination period, although the detection pattern varied among individuals. In Group 2-B, *p72*-targeted ASFV genomic DNA was detected in three of four animals during the vaccination period, whereas the animal that later succumbed to challenge showed no consistent *p72*-positive signal before challenge and remained negative at 28 dpv (Figure 6A; Table S8). In rectal and oral swab samples, *p72*-positive signals were detected intermittently during the vaccination and post-challenge periods, generally with higher Ct values than those observed in whole-blood samples (Figure 6C,E; Tables S9 and S10).

*I177L*-specific qPCR was negative in vaccinated animals at 28 dpv, immediately before challenge, whereas *I177L*-positive signals were detected after challenge in whole-blood and/or swab samples. After challenge, vaccinated survivors generally showed higher *I177L* Ct values, indicating lower levels of challenge virus-derived ASFV DNA, compared with positive-control animals. In contrast, the non-responder in Group 2-B showed lower *I177L* Ct values after challenge than the vaccinated survivors, consistent with a higher challenge virus-derived DNA burden. These findings suggest that vaccine-induced responses in protected animals contribute to better control of challenge virus-derived ASFV DNA after challenge (Figure 6B,D,F; Table S11).

**Figure 7:**
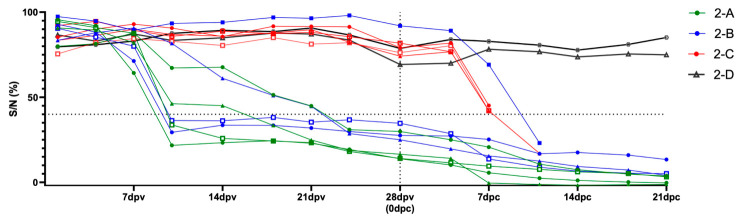
ASFV-specific antibody responses in serum samples from pigs in the second trial (10^5.0^ and 10^6.0^ TCID_50_/dose). Vaccinated pigs were orally administered ASFV-G-ΔI177L/ΔLVR at 10^5.0^ and 10^6.0^ TCID_50_/dose and subsequently challenged with ASFV-Hwacheon/2020 at 28 dpv. Green, blue, red, and gray lines represent groups 2-A, 2-B, 2-C, and 2-D, respectively. Different symbol shapes and fill patterns distinguish individual animals within each group. Each line represents an individual animal. The vertical dotted line indicates the day of challenge, corresponding to 28 dpv and 0 dpc. The horizontal dotted line indicates the assay cutoff, and S/N values ≤ 40% were considered positive.

In Group 2-A, all pigs achieved ASFV-specific p32 antibody levels exceeding the positive cutoff by 28 dpv, indicating complete seroconversion. In contrast, in Group 2-B, three out of four pigs reached the positive threshold by 28 dpv, while one animal remained seronegative throughout the study (Figure 7; Table S12). These *p72* qPCR and antibody response patterns were consistent with the post-challenge outcomes: the non-seroconverted animal in Group 2-B, which showed no consistent vaccine-associated *p72* detection before challenge, succumbed to challenge despite receiving the same high-dose oral vaccine as the other animals. In contrast, the remaining animals in the same group exhibited both *p72*-positive ASFV genomic DNA detection during the vaccination period and p32 antibody responses before challenge and survived.

### 3.3. Comparative Pathologic Evaluation for Lesions

Gross pathological lesions were evaluated comparatively among vaccinated, positive-control, and negative-control animals, and gross pathological scores were summarized for individual animals (Tables S15 and S16).

#### 3.3.1. Circulatory Organ Lesions

In Group 1-A (10^2.25^TCID_50_/dose), three out of four animals did not survive following ASFV challenge and exhibited severe gross lesions consistent with acute ASF. The most prominent lesion was marked splenomegaly with friability and dark discoloration, indicative of severe vascular congestion and lymphoid destruction commonly associated with acute ASFV infection. The lungs showed diffuse pulmonary hyperemia, reflecting systemic vascular damage and inflammatory responses induced by viral replication. In the kidneys, multifocal petechiae were observed in the renal cortex, suggesting hemorrhagic injury to the microvasculature. Additionally, dark discoloration of the liver and mild pericardial effusion was observed in some animals, further indicating systemic circulatory disturbances associated with severe infection. These gross lesions were comparable to those observed in the challenge control group, suggesting that the vaccine dose used in this group was insufficient to prevent systemic disease progression.

**Figure 8:**
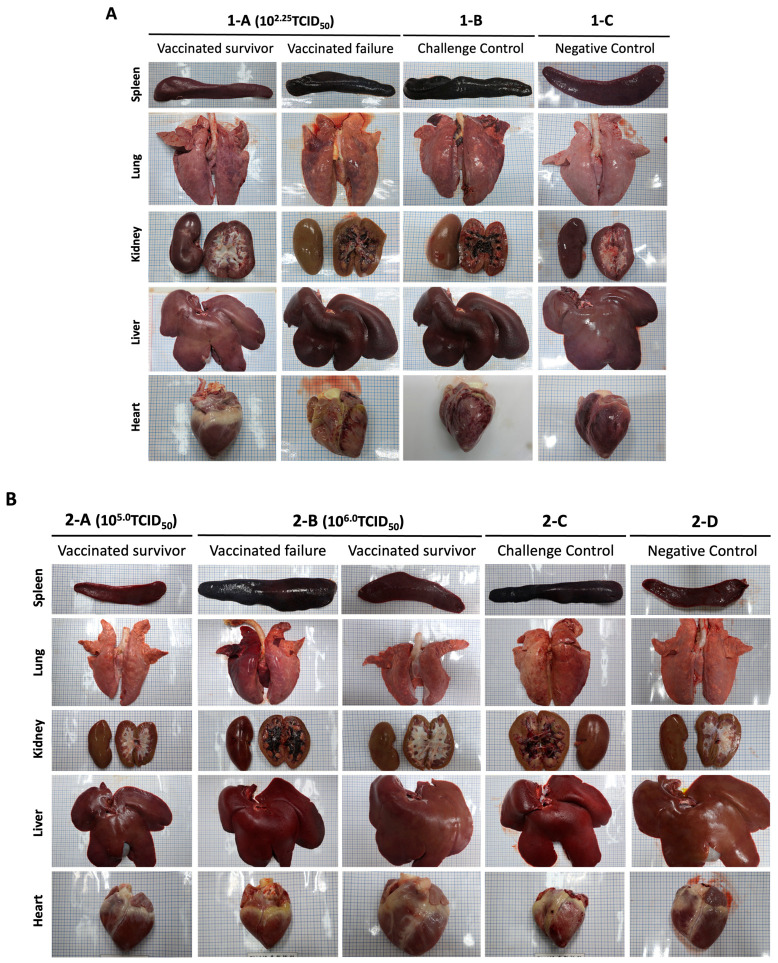
Gross pathological findings in major circulatory organs following vaccination and ASFV challenge. Representative gross lesions in the spleen, lung, kidney, liver and heart (top to bottom) collected at necropsy from pigs in different experimental groups. (**A**) Gross lesions from pigs vaccinated with ASFV-G-ΔI177L/ΔLVR at 10^2.25^ TCID_50_. (**B**) Gross lesions from pigs vaccinated with higher doses of ASFV-G-ΔI177L/ΔLVR (10^5.0^ and 10^6.0^ TCID_50_).

In contrast, the single surviving animal in this group exhibited only limited gross pathological changes and no severe hemorrhagic or necrotic lesions in major organs. (Figure 8A).

In Group 2-A (10^5.0^ TCID_50_/dose), all animals survived the ASFV challenge and exhibited either no or only mild gross lesions. Notably, no hemorrhagic or necrotic lesions were observed in major visceral organs such as the spleen, lungs, liver, kidneys, and heart. The preservation of normal organ morphology indicates that the mid-dose vaccine provided effective protection against the development of systemic vascular damage and severe pathological changes typically associated with acute ASFV infection.

Similarly, in Group 2-B (10^6.0^ TCID_50_/dose), three out of four animals survived the challenge and showed either no lesions or only mild gross pathological changes. Most major organs appeared grossly normal, with no evidence of hemorrhage or necrosis. However, compared with the animals of the challenge control group, the single non-surviving animal displayed severe lesions, including marked splenomegaly, renal petechiae, and pulmonary hyperemia—hallmarks of uncontrolled viral replication and progressive systemic disease. This outcome suggests that failure of vaccine uptake may occur even after administration of a high vaccine dose, leading to a lack of protection against virulent ASFV challenge (Figure 8B).

#### 3.3.2. Gross Pathology and Histopathology of Lymphoid Organs

To evaluate the impact of oral vaccination on lymphoid tissues, both gross and histopathological lesions were examined in the submandibular, inguinal, and mesenteric lymph nodes.

In Group 1-A (10^2^·^25^ TCID_50_/dose), the single surviving animal showed no remarkable gross lesions in any of the lymphoid tissues examined. Similarly, all animals in Group 2-A (10^5^·^0^ TCID_50_/dose) and the three surviving animals in Group 2-B (10^6^·^0^ TCID_50_/dose) exhibited no or only minimal changes in the lymph nodes, suggesting a protective effect of the vaccine. In contrast, animals that failed to develop protective immunity—such as those that died in Group 1-A (*n* = 3) and Group 2-B (*n* = 1)—showed severe lymphadenopathy characterized by hemorrhage, swelling, and dark discoloration of the mesenteric and peripheral lymph nodes. These lesions were very similar to those observed in the challenge control group, indicating a lack of immune protection in these individuals.

**Figure 9:**
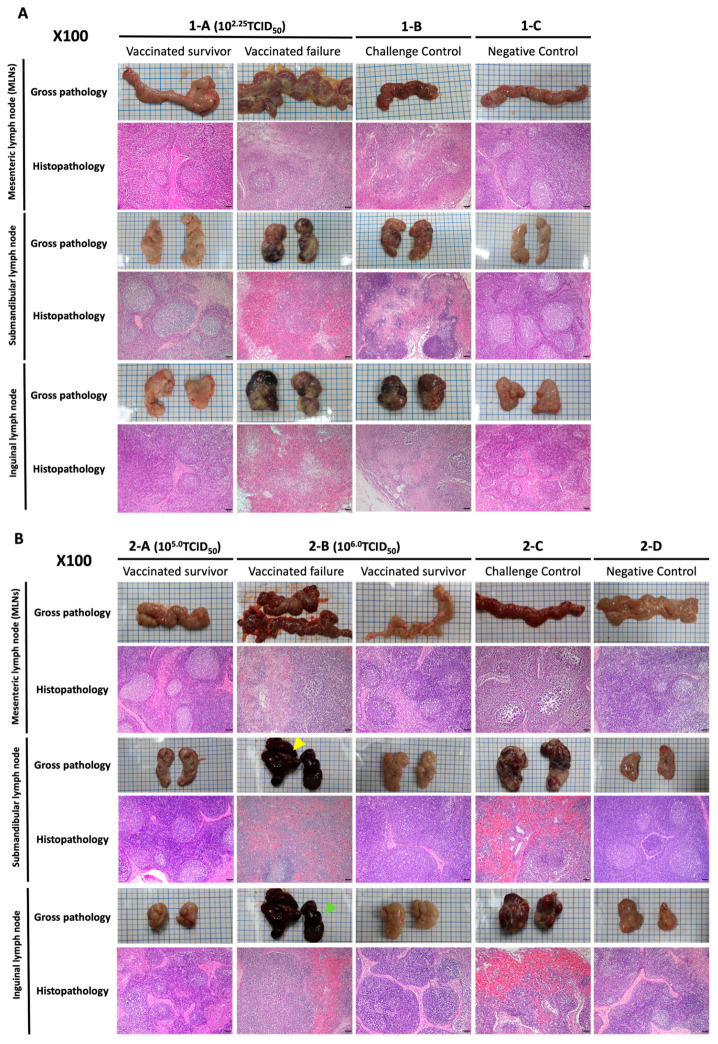
Gross and histopathological lesions in lymph nodes following vaccination and ASFV challenge. Representative gross pathological and histopathological findings in the mesenteric, inguinal, and submandibular lymph nodes (top to bottom) collected at necropsy from pigs in different experimental groups following vaccination and challenge with a homologous virulent ASFV strain. (**A**) Lymph node lesions from pigs vaccinated orally with ASFV-G-ΔI177L/ΔLVR at 10^2.25^ TCID_50_. (**B**) Lymph node lesions from pigs vaccinated orally with higher doses of ASFV-G-ΔI177L/ΔLVR (10^5^·^0^ and 10^6^·^0^ TCID_50_). Histopathological sections were stained with hematoxylin and eosin (H&E, ×100). Yellow arrows: inguinal lymph nodes; green arrows: submandibular lymph nodes.

Histopathological examination revealed a similar pattern. In animals that failed to develop protection following vaccination, the lymph nodes showed marked lymphoid depletion, characterized by reduced lymphocyte density and poorly defined follicular architecture. In addition, diffuse hemorrhage and hemorrhagic lymphadenitis were observed in some cases, indicating severe disruption of lymphoid tissue structure. These pathological changes were particularly evident in the submandibular and inguinal lymph nodes and closely resembled the lesions observed in the challenge control animals. In contrast, lymph nodes from vaccinated animals that survived the challenge largely preserved their lymphoid architecture, with clearly defined follicles and minimal evidence of hemorrhage or tissue destruction (Figure 9).

#### 3.3.3. ASFV Genomic DNA Detection in Tissue Homogenates

ASFV genomic DNA was also detected in tissue homogenates collected at necropsy. In the first trial, the surviving vaccinated animal in Group 1-A still showed detectable ASFV DNA in multiple tissues; however, the Ct values were generally higher than those observed in non-surviving vaccinated animals and positive-control animals, indicating a lower tissue viral DNA burden. Similarly, in the second trial, vaccinated animals that survived challenge showed detectable ASFV DNA in several tissues, but their overall Ct values tended to be higher than those of the positive-control animals and the non-surviving animal in Group 2-B. These results indicate that oral vaccination did not completely prevent systemic viral DNA detection after challenge but was generally associated with a lower tissue viral DNA burden in surviving vaccinated animals and protection from fatal disease (Tables S13 and S14).

## 4. Discussion

Despite substantial progress in ASF vaccine research, the development of a safe, effective, and field-applicable vaccine remains a major challenge [20,21]. Given the role of wild boar in ASF maintenance and transmission, oral vaccination represents a practical strategy for vaccine delivery in free-ranging populations [22,23]. Therefore, this study evaluated the safety, immunogenicity, and protective efficacy of orally administered ASFV-G-ΔI177L/ΔLVR in pigs.

Whole-genome sequencing of serially passaged ASFV-G-ΔI177L/ΔLVR showed limited sequence variation over the analyzed in vitro passage range, with no progressive accumulation of SNPs and no detected INDELs. These findings support the genetic stability of the vaccine candidate during cell-culture passage. However, because in vitro genetic stability does not fully exclude the possibility of additional changes after replication in vivo, further studies evaluating in vivo serial passage, contact transmission, and reversion-to-virulence risk are warranted.

In our previous studies, the ASFV-G-ΔI177L/ΔLVR vaccine candidate demonstrated complete protective efficacy following intramuscular administration at a dose of 10^2.25^ TCID_50_/dose, with 100% survival observed after challenge [7]. By comparison, oral administration in the present study showed different protective outcomes among the tested dose groups. The lowest dose (10^2.25^ TCID_50_/dose) was insufficient to consistently induce protection, whereas the intermediate dose (10^5.0^ TCID_50_/dose) conferred complete protection in all vaccinated pigs. Although the highest dose (10^6.0^ TCID_50_/dose) might be expected to provide comparable or greater protection, one animal in this group did not survive challenge. Therefore, the present data do not allow a definitive conclusion regarding the dose–response relationship of oral vaccination. This limitation is partly related to the small number of animals in each group, which was restricted by the high-containment ABL-3 conditions required for ASFV animal experiments. Accordingly, the protective outcomes observed in this study should be interpreted with caution, and further studies with larger group sizes and more controlled oral delivery conditions are required to determine the optimal oral vaccine dose and to clarify the relationship between administered dose, vaccine uptake, immune response, and protective efficacy.

Previous studies of orally administered ASFV LAV candidates have also shown that protection is strongly associated with successful seroconversion or immune induction, whereas non-responding animals may remain susceptible to challenge [23,24,25]. Consistent with these observations, the unprotected pigs in the present study, including the non-responder in the high-dose group, showed no consistent *p72*-targeted ASFV genomic DNA or p32 antibody responses before challenge. Their histopathological lesions were largely comparable to those of the positive-control animals. These findings suggest that the unprotected pigs likely succumbed to infection with the virulent challenge strain in the absence of detectable vaccine-induced responses.

The detection of ASFV DNA after challenge in some vaccinated survivors indicates that protection was not sterilizing. *I177L*-specific qPCR further supported that post-challenge ASFV DNA detection was associated, at least in part, with the *I177L*-containing challenge virus rather than only with the *I177L*-vaccine strain. However, the generally higher Ct values observed in protected animals, compared with positive-control or non-surviving vaccinated animals, suggest that vaccination reduced viral DNA burden in blood and tissues and contributed to protection from fatal ASF.

Oral vaccine delivery is inherently influenced by gastrointestinal stability, mucosal uptake efficiency, and host-dependent biological variability [26], which may contribute to variable protective outcomes. Therefore, incomplete vaccine uptake or insufficient early replication after oral administration may have contributed to the lack of immune induction in the unprotected animals. In addition, previous field studies using oral live vaccines against classical swine fever and rabies in wild boar and rabies in wildlife populations have demonstrated that bait uptake, age-dependent consumption, repeated distribution, and long-term surveillance are critical determinants of field efficacy [14,23,27,28,29,30,31]. These findings support the need to consider variability in oral vaccine uptake when interpreting the protective outcomes observed in the present study.

Although p32 ELISA provided useful information on vaccine-induced antibody responses, humoral responses alone may not fully explain the protective mechanisms induced by oral vaccination. Future studies should include PBMC-based assays, such as IFN-γ ELISpot, intracellular cytokine staining, or flow cytometric analysis of T-cell subsets, to better define immune correlates of protection.

Although the present study was conducted in domestic pigs under controlled experimental conditions, the results provide useful evidence supporting the feasibility of oral ASFV-G-ΔI177L/ΔLVR vaccination. In this study, the vaccine candidate was administered directly into the oral cavity as a liquid virus suspension, allowing the protective potential of oral delivery to be evaluated under controlled conditions before further development of bait-based formulations. Similarly, the intramuscular challenge model provided a standardized and stringent assessment of protective efficacy, although natural ASFV exposure in the field is more likely to occur through oro-nasal routes, environmental contamination, or contact transmission. Therefore, the next step toward practical application will be to optimize vaccine delivery for wild boar, including bait formulation, uptake efficiency, and field-relevant challenge models such as oro-nasal or contact exposure. These studies will be important to determine whether the protective efficacy observed under controlled conditions can be translated into effective oral vaccination strategies for free-ranging wild boar populations.

## 5. Conclusions

In this study, oral administration of ASFV-G-ΔI177L/ΔLVR induced protective immunity in pigs under the present experimental conditions. The 10^5.0^ TCID_50_/dose group showed the most consistent protection against virulent ASFV challenge, accompanied by stable rectal temperatures, absence of severe clinical disease, seroconversion, and reduced post-challenge viral DNA burden compared with positive-control or non-surviving vaccinated animals. Although a strictly dose-dependent protective pattern was not observed, these findings support the feasibility of oral immunization with ASFV-G-ΔI177L/ΔLVR and highlight the importance of optimizing vaccine uptake and delivery. Overall, ASFV-G-ΔI177L/ΔLVR represents a promising oral live attenuated vaccine candidate for further development. Future studies using bait-based formulations, larger animal numbers, and field-relevant challenge models will be important for advancing this candidate toward practical application, particularly in wild boar populations.

## Figures and Tables

**Table 1:** Summary of the vaccine trial design.

Trial	Group	No. of Pigs	Vaccine (Dose)	Challenge	Route (Vaccine/Challenge)
1st	1-A	4	ASFV-G-ΔI177L/ΔLVR (10^2.25^ TCID_50_/dose)	Hwacheon/2020 (Genotype-II)	Oral/IM
	1-B (Positive control)	4	-	Hwacheon/2020 (Genotype-II)	-/IM
	1-C (Negative control)	4	-	-	-/-
2nd	2-A	4	ASFV-G-ΔI177L/ΔLVR (10^5.0^ TCID_50_/dose)	Hwacheon/2020 (Genotype-II)	Oral/IM
	2-B	4	ASFV-G-ΔI177L/ΔLVR (10^6.0^ TCID_50_/dose)	Hwacheon/2020 (Genotype-II)	Oral/IM
	2-C (Positive control)	4	-	Hwacheon/2020 (Genotype-II)	-/IM
	2-D (Negative control)	2	-	-	-/-

IM, intramuscular immunization; Oral, oral immunization.

**Table 2:** Summary of SNPs and INDELs detected in the ASF vaccine candidate during serial passage.

Passage	Total SNPs	Intergenic SNPs	CDS SNPs	Nonsynonymous	Synonymous	Stop-Gained SNPs	INDELs	Affected Genes
P19	9	2	7	4	3	0	0	B263R (1), E119L (2), mCherry (4)
P24	5	1	4	4	0	0	0	B263R (1), D205R (1), E119L (2)
P29	5	1	4	4	0	0	0	B263R (1), D205R (1), E119L (2)

## Data Availability

The data and questionnaires that support the findings of this study are available from the corresponding author upon reasonable request.

## Supplementary Materials

The following supporting information can be downloaded at: https://www.mdpi.com/article/10.3390/vaccines14070609/s1, Table S1: Primer and probe sequences and oligonucleotide parameters used for the I177L-specific qPCR assays.; Table S2: Sequencing and mapping quality metrics for serially passaged vaccine virus samples; Table S3: Raw *p72* qPCR Ct values of ASFV genomic DNA in whole-blood samples from pigs in the first trial (10^2.25^ TCID_50_/dose); Table S4: Raw *p72* qPCR Ct values of ASFV genomic DNA in rectal swab samples from pigs in the first trial (10^2.25^ TCID_50_/dose); Table S5: Raw *p72* qPCR Ct values of ASFV genomic DNA in oral swab samples from pigs in the first trial (10^2.25^ TCID_50_/dose); Table S6: Raw *I177L*-specific differential qPCR Ct values in whole-blood, rectal swab, and oral swab samples from pigs in the first trial (10^2.25^ TCID_50_/dose), following oral vaccination with ASFV-G-ΔI177L/ΔLVR and challenge with ASFV-Hwacheon/2020; Table S7: Raw antibody response data in serum samples from pigs in the first trial following oral vaccination with 10^2.25^ TCID_50_/dose ASFV-G-ΔI177L/ΔLVR and challenge with ASFV-Hwacheon/2020; Table S8: Raw *p72* qPCR Ct values of ASFV genomic DNA in whole-blood samples from pigs in the second trial (10^5.0^ and 10^6.0^ TCID_50_/dose), following oral vaccination with ASFV-G-ΔI177L/ΔLVR and challenge with ASFV-Hwacheon/2020; Table S9: Raw *p72* qPCR Ct values of ASFV genomic DNA in rectal swab samples from pigs in the second trial (10^5.0^ and 10^6.0^ TCID_50_/dose), following oral vaccination with ASFV-G-ΔI177L/ΔLVR and challenge with ASFV-Hwacheon/2020; Table S10: Raw *p72* qPCR Ct values of ASFV genomic DNA in oral swab samples from pigs in the second trial (10^5.0^ and 10^6.0^ TCID_50_/dose), following oral vaccination with ASFV-G-ΔI177L/ΔLVR and challenge with ASFV-Hwacheon/2020; Table S11: Raw *I177L*-specific differential qPCR Ct values in whole-blood, rectal swab, and oral swab samples from pigs in the second trial (10^5.0^ and 10^6.0^ TCID_50_/dose), following oral vaccination with ASFV-G-ΔI177L/ΔLVR and challenge with ASFV-Hwacheon/2020; Table S12: Raw antibody response data in serum samples from pigs in the second trial following oral vaccination with 10^5.0^, 10^6.0^ TCID_50_/dose ASFV-G-ΔI177L/ΔLVR and challenge with ASFV-Hwacheon/2020; Table S13: Raw *p72* qPCR Ct values of ASFV genomic DNA in tissue homogenates collected at necropsy from pigs in the first trial; Table S14: Raw *p72* qPCR Ct values of ASFV genomic DNA in tissue homogenates collected at necropsy from pigs in the second trial; Table S15: Gross pathological scores of individual pigs in the first trial (10^2.25^ TCID_50_/dose) following challenge with ASFV-Hwacheon/2020; Table S16: Gross pathological scores of individual pigs in the second trial (10^5.0^ and 10^6.0^ TCID_50_/dose) following challenge with ASFV-Hwacheon/2020.

## Abbreviations

The following abbreviations are used in this manuscript:

ASFV	African swine fever virus
ABL-3	Animal biosafety level 3
DPV	Days post-vaccination
DPC	Days post-challenge
LAVs	Live attenuated vaccines
PIPEC	Plum Island porcine epithelial cell
EDTA	Ethylene diamine tetraacetic acid
WOAH	World Organization for Animal Health
OD	Optical density
