# Supplementary material for: Evaluation of Safety, Immunogenicity, and Protective Efficacy of an Orally Administered African Swine Fever Vaccine Candidate ASFV-G-∆I177L/∆LVR
Source: Vaccines (Basel). 2026 Jul 10;14(7):609. doi: 10.3390/vaccines14070609 (PMC13417075; doi:10.3390/vaccines14070609)
Supplement: Supplementary file 1 [file vaccines-14-00609-s001.zip › vaccines-4360853-supplementary.pdf]

Table S1. *Primer and probe sequences and oligonucleotide parameters used for the I177L-specific qPCR assays.*

qPCR	Primers and probes	Sequence 5′-3′	Position in the reference strain	sense	Tm (℃)	GC (%)	Dimer			
							Hairpin	Forward	Reverse	probe
I177L	Forward	5′GAACTGGAAAAAACTTTAACGGC3′	175529-175551	Positive	61.20	39.10	0.00	-4.00	-5.01	-4.84
	Reverse	5′CCATTACCGGCAACTAGG3′	175606-175588	Negative	62.10	57.90	0.00		-7.85	-4.08
	Probe	5′6FAM-ACGGATCCCCCTTCGCATTTGA-MGB-NFQ-3	175580-175559	Negative	68.00	54.50	-0.10			-7.16

Table S2. Sequencing and mapping quality metrics for serially passaged vaccine virus samples.

Passage	Total Reads (Millions)	GC content (%)	Duplicate reads (%)	Quality status (FastQC)	Mapping rate (%)	Mean sequencing depth	Covered bases	Genome coverage (%)
P19	10.5	45.0	88.6	PASS	94.4	5528.52	180,831	100
P24	21.5	39.0	90.9	PASS	98.1	16356.8	180,831	100
P29	24	40.0	90.6	PASS	98.16	18637.6	180,831	100

Table S3. Raw *p72* qPCR Ct values of ASFV genomic DNA in whole-blood samples from pigs in the first trial ($10^{2.25}$ $TCID_{50}$/dose).

Group		Ct* (qPCR, *p72*, Whole blood)												
		DPV (days post-vaccination)									DPC (days post-challenge)			
		0	4	7	10	14	18	21	25	28	4	7	11	14
1-A ($10^{2.25}$ $TCID_{50}$)	1-A1	45.00	45.00	45.00	45.00	45.00	45.00	45.00	45.00	45.00	16.19	13.32	D**	-
	1-A2	45.00	45.00	45.00	45.00	45.00	45.00	45.00	45.00	45.00	15.24	12.96	D	-
	1-A3†	45.00	45.00	45.00	45.00	33.57	25.13	26.81	26.92	30.85	30.04	30.96	27.20	20.28
	1-A4	45.00	45.00	45.00	45.00	45.00	45.00	45.00	45.00	45.00	18.31	14.04	D	-
1-B (Positive control)	1-B1	45.00	45.00	45.00	45.00	45.00	45.00	45.00	45.00	45.00	16.56	13.51	D	-
	1-B2	45.00	45.00	45.00	45.00	45.00	45.00	45.00	45.00	45.00	16.56	15.32	D	-
	1-B3	45.00	45.00	45.00	45.00	45.00	45.00	45.00	45.00	45.00	17.09	14.11	D	-
	1-B4	45.00	45.00	45.00	45.00	45.00	45.00	45.00	45.00	45.00	16.35	14.12	D	-
1-C (Negative control)	1-C1	45.00	45.00	45.00	45.00	45.00	45.00	45.00	45.00	45.00	45.00	45.00	45.00	45.00
	1-C2	45.00	45.00	45.00	45.00	45.00	45.00	45.00	45.00	45.00	45.00	45.00	45.00	45.00
	1-C3	45.00	45.00	45.00	45.00	45.00	45.00	45.00	45.00	45.00	45.00	45.00	45.00	45.00
	1-C4	45.00	45.00	45.00	45.00	45.00	45.00	45.00	45.00	45.00	45.00	45.00	45.00	45.00

†Animal survived after challenge; *Ct values <40 were interpreted as ASFV DNA detected in this study; Ct values ≥40 were considered negative or below the detection limit.
**D, dead; -, sample not collected.

Table S4. Raw *p72* qPCR Ct values of ASFV genomic DNA in rectal swab samples from pigs in the first trial ($10^{2.25}$ $TCID_{50}$/dose).

Group		Ct* (qPCR, *p72*, Rectal swab)												
		DPV (days post-vaccination)									DPC (days post-challenge)			
		0	4	7	10	14	18	21	25	28	4	7	11	14
1-A ($10^{2.25}$ $TCID_{50}$)	1-A1	45.00	45.00	45.00	45.00	45.00	45.00	45.00	45.00	45.00	30.80	25.80	D**	-
	1-A2	45.00	45.00	45.00	45.00	45.00	45.00	45.00	45.00	45.00	30.20	27.40	D	-
	1-A3†	45.00	45.00	45.00	45.00	45.00	45.00	45.00	45.00	45.00	45.00	31.30	37.60	36.80
	1-A4	45.00	45.00	45.00	45.00	45.00	45.00	45.00	45.00	45.00	36.20	28.90	D	-
1-B (Positive control)	1-B1	45.00	45.00	45.00	45.00	45.00	45.00	45.00	45.00	45.00	39.30	27.40	D	-
	1-B2	45.00	45.00	45.00	45.00	45.00	45.00	45.00	45.00	45.00	36.60	26.80	D	-
	1-B3	45.00	45.00	45.00	45.00	45.00	45.00	45.00	45.00	45.00	37.40	29.90	D	-
	1-B4	45.00	45.00	45.00	45.00	45.00	45.00	45.00	45.00	45.00	37.60	24.90	D	-
1-C (Negative control)	1-C1	45.00	45.00	45.00	45.00	45.00	45.00	45.00	45.00	45.00	45.00	45.00	45.00	45.00
	1-C2	45.00	45.00	45.00	45.00	45.00	45.00	45.00	45.00	45.00	45.00	45.00	45.00	45.00
	1-C3	45.00	45.00	45.00	45.00	45.00	45.00	45.00	45.00	45.00	45.00	45.00	45.00	45.00
	1-C4	45.00	45.00	45.00	45.00	45.00	45.00	45.00	45.00	45.00	45.00	45.00	45.00	45.00

†Animal survived after challenge; *Ct values <40 were interpreted as ASFV DNA detected in this study; Ct values ≥40 were considered negative or below the detection limit. **D, dead; -, sample not collected.

Table S5. Raw *p72* qPCR Ct values of ASFV genomic DNA in oral swab samples from pigs in the first trial ($10^{2.25}$ $TCID_{50}$/dose).

Group		Ct* (qPCR, *p72*, Oral swab)												
		DPV (days post-vaccination)									DPC (days post-challenge)			
		0	4	7	10	14	18	21	25	28	4	7	11	14
1-A ($10^{2.25}$ $TCID_{50}$)	1-A1	45.00	45.00	45.00	45.00	45.00	45.00	45.00	45.00	45.00	45.00	26.00	D**	-
	1-A2	45.00	45.00	45.00	45.00	45.00	45.00	45.00	45.00	45.00	36.20	23.20	D	-
	1-A3†	45.00	45.00	45.00	45.00	45.00	45.00	45.00	45.00	45.00	34.10	28.40	31.80	31.50
	1-A4	45.00	45.00	45.00	45.00	45.00	45.00	45.00	45.00	45.00	36.70	28.70	D	-
1-B (Positive control)	1-B1	45.00	45.00	45.00	45.00	45.00	45.00	45.00	45.00	45.00	38.30	22.90	D	-
	1-B2	45.00	45.00	45.00	45.00	45.00	45.00	45.00	45.00	45.00	37.00	27.90	D	-
	1-B3	45.00	45.00	45.00	45.00	45.00	45.00	45.00	45.00	45.00	38.40	27.90	D	-
	1-B4	45.00	45.00	45.00	45.00	45.00	45.00	45.00	45.00	45.00	36.90	27.70	D	-
1-C (Negative control)	1-C1	45.00	45.00	45.00	45.00	45.00	45.00	45.00	45.00	45.00	45.00	45.00	45.00	45.00
	1-C2	45.00	45.00	45.00	45.00	45.00	45.00	45.00	45.00	45.00	45.00	45.00	45.00	45.00
	1-C3	45.00	45.00	45.00	45.00	45.00	45.00	45.00	45.00	45.00	45.00	45.00	45.00	45.00
	1-C4	45.00	45.00	45.00	45.00	45.00	45.00	45.00	45.00	45.00	45.00	45.00	45.00	45.00

†Animal survived after challenge; *Ct values <40 were interpreted as ASFV DNA detected in this study; Ct values ≥40 were considered negative or below the detection limit.
**D, dead; -, sample not collected.

Table S6. Raw *I177L*-specific differential qPCR Ct values in whole-blood, rectal swab, and oral swab samples from pigs in the first trial ($10^{2.25}$ $TCID_{50}$/dose), following oral vaccination with ASFV-G-ΔI177L/ΔLVR and challenge with ASFV-Hwacheon/2020.

Group		Ct* (qPCR, *I177L*)														
		DPC (days post-challenge)														
		Whole blood					Rectal swab					Oral swab				
		0	4	7	11	14	0	4	7	11	14	0	4	7	11	14
1-A ($10^{2.25}$ $TCID_{50}$)	1-A1	40.00	16.00	13.40	D**	-	40.00	40.00	25.60	D**	-	40.00	38.30	26.00	D**	-
	1-A2	40.00	15.10	13.60	D	-	40.00	30.10	26.90	D	-	40.00	35.60	23.00	D	-
	1-A3†	40.00	40.00	40.00	29.00	21.80	40.00	40.00	30.90	40.00	40.00	40.00	33.80	27.80	31.60	40.00
	1-A4	40.00	18.10	13.90	D	-	40.00	35.90	28.50	D	-	40.00	36.40	28.90	D	-
1-B (Positive control)	1-B1	40.00	15.50	14.50	D	-	40.00	37.20	26.10	D	-	40.00	37.70	21.50	D	-
	1-B2	40.00	15.40	13.50	D	-	40.00	36.10	25.30	D	-	40.00	37.70	26.60	D	-
	1-B3	40.00	15.90	13.70	D	-	40.00	36.70	28.10	D	-	40.00	36.60	26.60	D	-
	1-B4	40.00	14.90	15.10	D	-	40.00	36.30	23.30	D	-	40.00	36.70	26.40	D	-
1-C (Negative control)	1-C1	40.00	40.00	40.00	40.00	40.00	40.00	40.00	40.00	40.00	40.00	40.00	40.00	40.00	40.00	40.00
	1-C2	40.00	40.00	40.00	40.00	40.00	40.00	40.00	40.00	40.00	40.00	40.00	40.00	40.00	40.00	40.00
	1-C3	40.00	40.00	40.00	40.00	40.00	40.00	40.00	40.00	40.00	40.00	40.00	40.00	40.00	40.00	40.00
	1-C4	40.00	40.00	40.00	40.00	40.00	40.00	40.00	40.00	40.00	40.00	40.00	40.00	40.00	40.00	40.00

†Animal survived after challenge; *Ct values <40 were considered positive; Ct = 40 indicates negative or below the detection limit. **D, dead; -, sample not collected.

Table S7. Raw antibody response data in serum samples from pigs in the first trial following oral vaccination with $10^{2.25}$ $TCID_{50}$/dose ASFV-G-ΔI177L/ΔLVR and challenge with ASFV-Hwacheon/2020.

Group		S/N%*(cELISA)												
		DPV (days post-vaccination)									DPC (days post-challenge)			
		0	4	7	10	14	18	21	25	28	4	7	11	14
1-A ($10^{2.25}$ $TCID_{50}$)	1-A1	77.50	89.43	86.78	79.75	90.90	84.51	80.00	88.74	87.66	82.06	42.76	D**	-
	1-A2	83.37	85.11	92.52	79.63	87.17	92.97	96.32	88.28	87.02	90.81	52.50	D	-
	1-A3†	95.94	90.65	79.95	76.40	86.85	92.78	29.20	32.14	28.27	18.59	11.47	10.96	10.12
	1-A4	84.78	79.24	80.92	81.69	81.93	76.05	75.45	79.17	76.02	69.80	42.86	D	-
1-B (Positive control)	1-B1	89.10	82.27	85.62	81.69	76.78	68.87	73.52	74.30	68.99	67.72	13.45	3.09	D
	1-B2	91.42	86.01	68.73	71.82	78.94	80.05	76.60	83.17	67.76	78.00	36.68	D	-
	1-B3	90.14	81.88	68.86	78.98	83.22	81.43	77.01	65.77	62.07	68.76	13.54	8.96	D
	1-B4	72.99	74.60	68.15	59.96	57.01	59.82	61.89	58.69	64.11	65.15	12.26	5.22	D
1-C (Negative control)	1-C1	83.75	87.36	87.49	94.20	89.50	71.08	68.66	76.18	77.37	85.81	75.63	91.04	94.46
	1-C2	89.17	92.52	76.27	92.39	85.64	85.16	76.34	87.42	82.96	92.85	82.60	99.94	95.36
	1-C3	87.43	83.43	67.18	86.07	85.42	84.41	80.81	79.30	82.20	86.18	79.39	102.39	91.30
	1-C4	65.96	70.86	68.09	85.94	83.33	81.08	78.49	68.44	73.60	75.38	71.97	99.16	91.81

†Animal survived after challenge; *S/N% values ≤40% were considered positive. **D, dead; -, sample not collected.

Table S8. Raw *p72* qPCR Ct values of ASFV genomic DNA in whole-blood samples from pigs in the second trial ($10^{5.0}$ and $10^{6.0}$ $TCID_{50}$/dose), following oral vaccination with ASFV-G-ΔI177L/ΔLVR and challenge with ASFV-Hwacheon/2020.

Group		Ct*(qPCR, *p72*, Whole blood)														
		DPV (days post-vaccination)									DPC (days post-challenge)					
		0	4	7	10	14	18	21	25	28	4	7	11	14	18	21
2-A ($10^{5.0}$ $TCID_{50}$)	2-A1	45.00	38.14	18.19	21.80	21.55	23.99	22.37	24.72	25.49	27.81	28.20	30.80	30.50	29.77	30.92
	2-A2	45.00	45.00	36.77	38.99	39.90	45.00	45.00	41.45	45.00	31.94	24.35	26.61	26.23	24.66	25.61
	2-A3	45.00	45.00	38.06	30.21	32.06	36.21	37.14	38.97	39.58	32.26	27.31	31.25	35.13	36.26	35.82
	2-A4	45.00	40.02	21.07	20.18	21.19	21.75	22.41	25.22	24.69	26.67	28.67	32.04	32.74	31.76	32.27
2-B ($10^{6.0}$ $TCID_{50}$)	2-B1	45.00	39.99	34.72	28.18	31.05	32.92	33.90	36.20	36.47	35.63	22.69	26.65	26.18	25.94	25.96
	2-B2	45.00	45.00	45.00	40.59	33.17	34.33	30.06	29.61	29.84	33.09	33.56	37.21	36.75	30.73	31.40
	2-B3††	45.00	45.00	45.00	40.69	45.00	45.00	45.00	38.47	45.00	22.76	16.89	16.86	D	-	-
	2-B4	45.00	45.00	38.03	30.42	34.86	36.78	39.92	39.97	45.00	35.32	23.04	24.77	24.87	24.81	26.79
2-C (Positive control)	2-C1	45.00	45.00	45.00	45.00	45.00	45.00	45.00	39.98	45.00	17.66	16.76	D**	-	-	-
	2-C2	45.00	45.00	45.00	45.00	45.00	45.00	45.00	45.00	45.00	17.86	16.67	18.09	D	-	-
	2-C3	45.00	40.36	45.00	45.00	40.35	45.00	41.52	45.00	45.00	17.52	16.26	D	-	-	-
	2-C4	45.00	45.00	45.00	45.00	45.00	45.00	45.00	45.00	45.00	15.97	15.92	D	-	-	-
2-D (Negative control)	2-D1	45.00	45.00	45.00	45.00	45.00	45.00	45.00	45.00	45.00	45.00	40.63	45.00	45.00	45.00	45.00
	2-D2	45.00	45.00	45.00	45.00	45.00	45.00	45.00	45.00	45.00	45.00	45.00	45.00	45.00	45.00	45.00

†† Animal died after challenge; *Ct values <40 were interpreted as ASFV DNA detected in this study; Ct values ≥40 were considered negative or below the detection limit. **D, dead; -, sample not collected.

Table S9. Raw *p72* qPCR Ct values of ASFV genomic DNA in rectal swab samples from pigs in the second trial ($10^{5.0}$ and $10^{6.0}$ $TCID_{50}$/dose), following oral vaccination with ASFV-G-ΔI177L/ΔLVR and challenge with ASFV-Hwacheon/2020.

Group		Ct*(qPCR, *p72*, Rectal swab)														
		DPV (days post-vaccination)									DPC (days post-challenge)					
		0	4	7	10	14	18	21	25	28	4	7	11	14	18	21
2-A ($10^{5.0}$ $TCID_{50}$)	2-A1	45.00	45.00	37.63	36.19	45.00	45.00	39.63	34.78	39.50	45.00	38.10	45.00	45.00	45.00	39.66
	2-A2	45.00	45.00	45.00	45.00	45.00	40.02	45.00	45.00	45.00	45.00	37.91	39.11	45.00	45.00	45.00
	2-A3	45.00	45.00	39.95	45.00	45.00	38.94	45.00	40.57	45.00	38.74	40.08	45.00	45.00	45.00	45.00
	2-A4	45.00	45.00	39.47	45.00	38.46	38.38	45.00	45.00	45.00	45.00	39.25	45.00	45.00	45.00	40.39
2-B ($10^{6.0}$ $TCID_{50}$)	2-B1	45.00	45.00	45.00	39.16	45.00	45.00	45.00	45.00	45.00	45.00	40.41	39.30	36.05	38.84	45.00
	2-B2	45.00	45.00	45.00	45.00	45.00	45.00	45.00	41.96	45.00	40.88	38.43	39.55	36.01	39.96	38.87
	2-B3[††]	45.00	45.00	45.00	45.00	45.00	45.00	45.00	45.00	45.00	45.00	29.86	27.46	D	-	-
	2-B4	45.00	45.00	45.00	45.00	45.00	45.00	45.00	38.06	45.00	40.71	38.90	31.33	37.47	35.12	37.23
2-C (Positive control)	2-C1	45.00	45.00	45.00	45.00	39.59	40.02	45.00	39.91	45.00	30.93	28.17	D**	-	-	-
	2-C2	45.00	45.00	45.00	45.00	45.00	45.00	45.00	40.08	39.88	35.63	30.06	28.81	D	-	-
	2-C3	45.00	45.00	45.00	45.00	45.00	45.00	45.00	40.59	45.00	33.40	24.90	D	-	-	-
	2-C4	45.00	45.00	45.00	45.00	45.00	45.00	45.00	39.05	45.00	29.34	26.52	D	-	-	-
2-D (Negative control)	2-D1	45.00	45.00	45.00	45.00	45.00	45.00	45.00	45.00	45.00	45.00	45.00	45.00	45.00	45.00	45.00
	2-D2	45.00	45.00	45.00	45.00	45.00	45.00	45.00	45.00	45.00	45.00	45.00	45.00	45.00	45.00	45.00

[††] Animal died after challenge; *Ct values <40 were interpreted as ASFV DNA detected in this study; Ct values ≥40 were considered negative or below the detection limit. **D, dead; -, sample not collected.

Table S10. Raw *p72* qPCR Ct values of ASFV genomic DNA in oral swab samples from pigs in the second trial ($10^{5.0}$ and $10^{6.0}$ $TCID_{50}$/dose), following oral vaccination with ASFV-G-ΔI177L/ΔLVR and challenge with ASFV-Hwacheon/2020.

Group		Ct*(qPCR, *p72*, Oral swab)														
		DPV (days post-vaccination)									DPC (days post-challenge)					
		0	4	7	10	14	18	21	25	28	4	7	11	14	18	21
2-A ($10^{5.0}$ $TCID_{50}$)	2-A1	45.00	41.02	45.00	38.96	45.00	45.00	39.58	45.00	39.47	45.00	37.64	38.09	45.00	45.00	45.00
	2-A2	45.00	40.35	38.52	39.81	45.00	39.29	45.00	40.97	45.00	45.00	36.41	38.49	45.00	39.36	39.62
	2-A3	45.00	45.00	45.00	45.00	45.00	39.03	45.00	45.00	45.00	45.00	38.99	40.53	45.00	39.10	38.92
	2-A4	45.00	45.00	45.00	45.00	45.00	45.00	45.00	39.28	45.00	35.60	34.88	35.82	38.18	45.00	45.00
2-B ($10^{6.0}$ $TCID_{50}$)	2-B1	45.00	45.00	45.00	37.68	45.00	39.44	45.00	41.00	45.00	39.94	35.92	31.78	33.60	35.03	45.00
	2-B2	45.00	45.00	45.00	40.46	45.00	39.37	38.56	39.59	45.00	45.00	45.00	32.99	36.32	33.19	32.50
	2-B3††	45.00	39.63	45.00	45.00	45.00	45.00	45.00	45.00	45.00	45.00	32.94	28.87	D	-	-
	2-B4	45.00	45.00	45.00	45.00	45.00	36.27	38.92	45.00	45.00	45.00	38.24	29.07	36.89	38.54	35.02
2-C (Positive control)	2-C1	45.00	45.00	45.00	45.00	45.00	45.00	45.00	45.00	45.00	36.94	27.93	D**	-	-	-
	2-C2	45.00	45.00	45.00	45.00	45.00	45.00	45.00	45.00	45.00	38.37	29.15	27.21	D	-	-
	2-C3	45.00	45.00	45.00	39.72	45.00	45.00	45.00	38.50	45.00	37.38	26.72	D	-	-	-
	2-C4	45.00	45.00	45.00	45.00	45.00	45.00	40.47	39.58	45.00	37.58	25.72	D	-	-	-
2-D (Negative control)	2-D1	45.00	45.00	45.00	45.00	45.00	45.00	45.00	45.00	38.65	45.00	45.00	45.00	45.00	45.00	45.00
	2-D2	45.00	45.00	45.00	45.00	45.00	45.00	45.00	45.00	45.00	45.00	45.00	45.00	45.00	45.00	45.00

†† Animal died after challenge; *Ct values <40 were interpreted as ASFV DNA detected in this study; Ct values ≥40 were considered negative or below the detection limit. **D, dead; -, sample not collected.

Table S11. Raw *I177L*-specific differential qPCR Ct values in whole-blood, rectal swab, and oral swab samples from pigs in the second trial ($10^{5.0}$ and $10^{6.0}$ $TCID_{50}$/dose), following oral vaccination with ASFV-G-ΔI177L/ΔLVR and challenge with ASFV-Hwacheon/2020.

Group		Ct* (qPCR, *I177L*)																				
		DPC (days post-challenge)																				
		Whole blood							Rectal swab							Oral swab						
		0	4	7	11	14	18	21	0	4	7	11	14	18	21	0	4	7	11	14	18	21
2-A ($10^{5.0}$ $TCID_{50}$)	2-A1	40.00	40.00	40.00	40.00	40.00	40.00	40.00	40.00	40.00	35.87	40.00	40.00	40.00	40.00	40.00	40.00	38.07	38.37	40.00	40.00	40.00
	2-A2	40.00	32.50	23.91	25.22	29.74	25.45	24.74	40.00	40.00	36.50	39.40	40.00	40.00	40.00	40.00	40.00	34.76	36.07	40.00	37.29	40.00
	2-A3	40.00	36.78	28.53	32.11	40.00	35.73	35.57	40.00	40.00	40.00	40.00	40.00	40.00	40.00	40.00	40.00	37.07	35.11	40.00	40.00	40.00
	2-A4	40.00	40.00	40.00	40.00	40.00	32.11	33.12	40.00	40.00	40.00	40.00	40.00	40.00	40.00	40.00	33.92	32.52	33.38	35.37	40.00	40.00
2-B ($10^{6.0}$ $TCID_{50}$)	2-B1	40.00	40.00	20.45	25.24	27.81	24.29	24.76	40.00	40.00	36.99	33.69	33.47	38.01	40.00	40.00	40.00	35.15	29.39	31.45	33.56	40.00
	2-B2	40.00	40.00	40.00	40.00	35.02	29.23	30.93	40.00	40.00	37.61	37.29	33.39	37.19	36.22	40.00	40.00	36.94	30.74	33.71	31.14	30.90
	2-B3††	40.00	21.69	16.71	15.16	D	-	-	40.00	40.00	28.12	25.87	D	-	-	40.00	40.00	31.46	26.64	D		
	2-B4	40.00	37.18	40.00	23.19	24.61	37.16	27.13	40.00	40.00	35.05	29.43	34.94	33.45	34.50	40.00	40.00	36.04	26.97	34.00	37.72	33.24
2-C (Positive control)	2-C1	40.00	17.17	14.62	D**	-	-	-	40.00	29.16	26.27	D	-	-	-	40.00	35.07	26.30	D	-	-	-
	2-C2	40.00	16.10	15.32	16.73	D	-	-	40.00	33.17	28.43	26.89	D	-	-	40.00	37.21	27.46	25.95	D	-	-
	2-C3	40.00	16.44	15.60	D	-	-	-	40.00	31.38	22.92	D	-	-	-	40.00	36.93	24.73	D	-	-	-
	2-C4	40.00	14.86	14.45	D	-	-	-	40.00	27.60	24.24	D	-	-	-	40.00	35.37	23.84	D	-	-	-
2-D (Negative control)	2-D1	40.00	40.00	40.00	40.00	40.00	40.00	40.00	40.00	40.00	40.00	40.00	40.00	40.00	40.00	40.00	40.00	40.00	40.00	40.00	40.00	40.00
	2-D2	40.00	40.00	40.00	40.00	40.00	40.00	40.00	40.00	40.00	40.00	40.00	40.00	40.00	40.00	40.00	40.00	40.00	40.00	40.00	40.00	40.00

†† Animal died after challenge; *Ct values <40 were considered positive; Ct = 40 indicates negative or below the detection limit. **D, dead; -, sample not collected.

Table S12. Raw antibody response data in serum samples from pigs in the second trial following oral vaccination with $10^{5.0}$, $10^{6.0}$ $TCID_{50}$/dose ASFV-G-ΔI177L/ΔLVR and challenge with ASFV-Hwacheon/2020.

Group		S/N%*(cELISA)														
		DPV (days post-vaccination)									DPC (days post-challenge)					
		0	4	7	10	14	18	21	25	28	4	7	11	14	18	21
2-A ($10^{5.0}$ $TCID_{50}$)	2-A1	95.55	91.99	64.23	21.75	23.29	24.45	22.93	19.32	13.85	10.20	5.60	2.40	1.10	0.10	-0.30
	2-A2	90.69	89.44	81.97	46.21	45.07	33.42	24.87	18.75	16.47	14.10	-0.40	-1.30	-1.70	-1.40	-1.20
	2-A3	79.89	81.38	87.96	67.18	67.63	51.36	44.86	30.94	29.86	25.00	20.70	10.70	7.40	4.80	3.70
	2-A4	94.54	90.87	87.60	33.67	25.81	24.24	23.24	18.12	14.02	11.40	9.50	7.60	6.20	5.20	3.20
2-B ($10^{6.0}$ $TCID_{50}$)	2-B1	92.82	88.26	71.29	29.38	33.58	33.47	31.90	29.86	27.52	27.20	25.20	16.80	17.50	16.00	13.40
	2-B2	83.45	87.90	90.69	81.64	61.07	51.05	44.75	28.66	25.01	19.60	15.50	12.50	9.30	7.20	4.10
	2-B3††	97.39	94.72	89.38	93.39	93.97	96.90	96.41	98.06	91.97	89.10	69.10	23.10	D	-	-
	2-B4	90.45	85.35	80.00	36.27	36.15	38.14	35.38	36.70	34.70	28.60	13.70	8.90	6.60	5.40	5.10
2-C (Positive control)	2-C1	85.29	90.09	92.94	90.68	85.57	91.71	91.55	91.34	79.03	81.80	45.10	D**	-	-	-
	2-C2	83.45	86.36	89.92	86.89	85.89	87.83	89.66	82.34	74.13	76.70	42.10	17.00	D	-	-
	2-C3	92.11	94.54	89.21	85.76	88.77	87.30	88.14	85.53	81.77	76.70	42.10	D	-	-	-
	2-C4	75.50	82.09	84.40	82.82	80.38	85.05	81.22	81.99	76.24	80.30	42.10	D	-	-	-
2-D (Negative control)	2-D1	86.60	82.98	87.12	83.39	84.94	87.67	87.12	83.53	69.23	70.00	78.20	76.70	73.70	75.40	74.90
	2-D2	79.60	80.84	82.99	87.51	89.30	88.56	90.66	86.55	78.69	83.90	82.80	80.60	77.70	81.00	85.10

†† Animal died after challenge; *S/N% values ≤40% were considered positive. **D, dead; -, sample not collected.

Table S13. Raw *p72* qPCR Ct values of ASFV genomic DNA in tissue homogenates collected at necropsy from pigs in the first trial.

Trial	Group	Name	Submandibular LN	Inguinal LN	Mesenteric LN	Tonsil	Heart	Kidney	Lung	Spleen	Liver
1st	1-A ($10^{2.25}$ $TCID_{50}$)	1-A-1	17.27	18.52	21.08	19.06	21.85	21.92	20.97	17.19	18.91
		1-A-2	15.70	15.69	16.46	16.65	21.54	14.00	17.97	20.70	18.54
		1-A-3†	23.58	27.78	27.46	21.70	32.59	29.91	27.58	23.09	27.01
		1-A-4	17.44	17.77	19.21	18.48	22.98	21.50	18.12	16.81	17.66
	1-B (Positive control)	1-B-1	16.35	16.81	19.31	18.36	17.63	19.78	17.60	15.38	17.21
		1-B-2	17.05	17.44	17.43	17.49	23.94	20.60	17.86	15.37	16.86
		1-B-3	16.17	15.85	17.22	16.51	19.29	19.26	15.95	14.45	16.14
		1-B-4	14.94	16.71	18.38	18.11	22.77	20.69	17.42	14.43	15.78
	1-C (Negative control)	1-C-1	45.00	45.00	45.00	45.00	45.00	45.00	45.00	45.00	45.00
		1-C-2	45.00	45.00	45.00	45.00	45.00	45.00	45.00	45.00	45.00
		1-C-3	45.00	45.00	45.00	45.00	45.00	45.00	45.00	45.00	45.00
		1-C-4	45.00	45.00	45.00	45.00	45.00	45.00	45.00	45.00	45.00

† Animal survived after challenge; Ct values <40 were interpreted as ASFV DNA detected in this study; Ct values ≥40 were considered negative or below the detection limit. LN, lymph node.

Table S14. Raw *p72* qPCR Ct values of ASFV genomic DNA in tissue homogenates collected at necropsy from pigs in the second trial.

Trial	Group	Name	Submandibular LN	Inguinal LN	Mesenteric LN	Tonsil	Heart	Kidney	Lung	Spleen	Liver
2nd	2-A ($10^{5.0}$ $TCID_{50}$)	2-A-1	27.46	33.38	38.01	34.68	38.99	38.34	37.52	36.54	37.30
		2-A-2	32.39	35.21	36.79	30.77	35.55	34.66	34.88	31.33	34.66
		2-A-3	30.86	36.61	38.07	28.01	37.48	39.71	34.35	36.38	38.97
		2-A-4	28.69	28.57	33.98	35.87	45.00	35.95	29.72	29.97	28.97
	2-B ($10^{6.0}$ $TCID_{50}$)	2-B-1	23.84	26.54	33.76	23.94	36.66	33.65	33.00	30.98	32.35
		2-B-2	21.30	23.00	27.57	19.77	37.23	33.17	19.18	28.56	31.27
		2-B-3††	21.07	21.02	24.06	23.02	26.38	23.85	21.99	19.90	21.82
		2-B-4	27.38	28.08	33.63	29.39	40.25	38.14	27.94	32.09	36.46
	2-C (Positive control)	2-C-1	17.46	17.67	18.48	18.76	22.73	22.78	20.02	17.14	18.98
		2-C-2	20.44	21.74	22.99	22.48	23.15	22.94	21.47	18.46	19.33
		2-C-3	16.46	16.82	18.42	22.41	21.89	20.30	18.16	16.86	16.95
		2-C-4	17.54	17.56	20.88	20.01	23.62	20.92	18.82	16.82	19.01
	2-D (Negative control)	2-D-1	45.00	45.00	45.00	45.00	45.00	45.00	45.00	45.00	45.00
		2-D-2	45.00	45.00	45.00	45.00	45.00	45.00	45.00	45.00	45.00

†† Animal died after challenge; Ct values <40 were interpreted as ASFV DNA detected in this study; Ct values ≥40 were considered negative or below the detection limit. LN, lymph node.

Table S15. Gross pathological scores of individual pigs in the first trial ($10^{2.25}$ $TCID_{50}$/dose) following challenge with ASFV-Hwacheon/2020.

Trial	Group	Name	Total score	Body condition	Integument	Spleen	Lung	Kidney	Liver	Cardiovascular changes	Lymphoid tissue
1st	1-A ($10^{2.25}$ $TCID_{50}$)	1-A-1	16	1	2	3	3	2	1	1	3
		1-A-2	13	1	2	3	1	2	1	0	3
		1-A-3†	6	0	0	0	2	1	1	1	1
		1-A-4	20	1	2	3	3	3	3	2	3
	1-B (Positive control)	1-B-1	20	1	2	3	3	3	2	3	3
		1-B-2	20	1	2	3	3	3	3	2	3
		1-B-3	16	1	2	2	3	2	1	3	2
		1-B-4	16	1	2	3	2	2	3	1	2
	1-C (Negative control)	1-C-1	1	0	0	0	0	0	0	1	0
		1-C-2	1	0	0	0	0	0	0	1	0
		1-C-3	0	0	0	0	0	0	0	0	0
		1-C-4	2	0	0	0	0	0	0	1	1

† Animal survived after challenge.

Table S16. Gross pathological scores of individual pigs in the second trial ($10^{5.0}$ and $10^{6.0}$ $TCID_{50}$/dose) following challenge with ASFV-Hwacheon/2020.

Trial	Group	Name	Total score	Body condition	Integument	Spleen	Lung	Kidney	Liver	Cardiovascular changes	Lymphoid tissue
2nd	2-A ($10^{5.0}$ $TCID_{50}$)	2-A-1	1	0	0	0	1	0	0	0	0
		2-A-2	0	0	0	0	0	0	0	0	0
		2-A-3	1	0	0	0	0	1	0	0	0
		2-A-4	1	0	0	0	0	0	0	0	1
	2-B ($10^{6.0}$ $TCID_{50}$)	2-B-1	2	0	0	0	0	0	0	1	1
		2-B-2	1	0	0	0	1	0	0	0	0
		2-B-3††	18	1	2	2	3	3	2	2	3
		2-B-4	0	0	0	0	0	0	0	0	0
	2-C (Positive control)	2-C-1	19	1	2	3	3	3	2	2	3
		2-C-2	19	1	2	3	3	3	2	2	3
		2-C-3	19	1	2	3	3	3	2	2	3
		2-C-4	20	1	2	3	3	3	3	2	3
	2-D (Negative control)	2-D-1	0	0	0	0	0	0	0	0	0
		2-D-2	1	0	0	0	0	0	0	0	1

†† Animal died after challenge
